# Supplementary material for: Resonant terahertz detection using graphene plasmons
Source: Nat Commun. 2018 Dec 19;9:5392. doi: 10.1038/s41467-018-07848-w (PMC6300605; doi:10.1038/s41467-018-07848-w)
Supplement: Supplementary file 1 — Supplementary Information [file 41467_2018_7848_MOESM1_ESM.pdf]

Supplementary information for
Resonant terahertz detection using graphene plasmons

D.A. Bandurin, D. Svintsov et al.

Supplementary Note 1: Device characterization

Our photodetectors represent two-terminal field-effect transistors (FET) and, therefore, the measured conductance (Fig. 1d of the main text), which contains non-zero contribution from the BLG-metal contact, does not provide the information on the quality of the FET channel. In order to estimate the mobility of charge carriers in the BLG channel, we fabricated a reference multi-terminal Hall bar using the same procedure as described in Methods. The Hall bar was characterized using the standard four-terminal geometry that involved the measurements of its sheet resistance ρ as a function of carrier density n and temperature T (Supplementary Fig. 1). One can see a typical field-effect behavior for high-quality graphene that manifests itself in sharp peak in ρ at the charge neutrality point which decays steeply with increasing n. The charge carrier mobility μ was calculated using the Drude formula, $\mu = \sigma/ne$, and for typical $n = 10^{12}$ cm^{-2} exceeded 10 m^2/Vs at liquid helium T and remained around 2.5 m^2/Vs at room temperature.

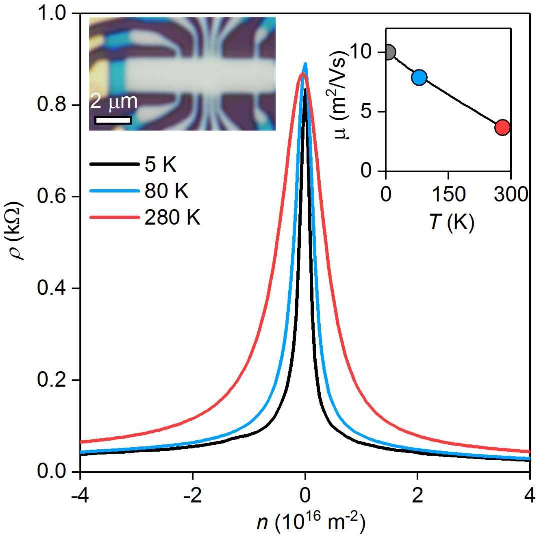

Supplementary Figure 1. **Reference multiterminal BLG field effect transistor.** Sheet resistance as a function of n for different T measured in the standard four-terminal geometry. Left inset: Optical photographs of our reference Hall bar. Right inset: Mobility as a function of T measured at $n = 10^{12}$ cm^{-2}.

Supplementary Note 2: High-responsivity THz detection in dual-gated BLG field effect transistors

As discussed in the main text, the responsivity of the THz detectors made of the field effect transistors is proportional to the sensitivity of the FET conductivity to the gate voltage variation. To improve the performance of our detectors, we took advantage of BLG's gate-tunable band structure and fabricated a dual-gated device (top and bottom insets of Supplementary Fig. 2a). The idea is that when an electric field is applied perpendicular to the channel it induces a band gap in BLG that leads to a steeper dependence of the FET resistance R_{2pt} on the gate voltage. Supplementary Fig. 2a shows examples of $R_{2pt}(V_{tg})$ dependences measured at few V_{bg} demonstrating the expected increase of R_{2pt} with increasing the average displacement field $D = \frac{\varepsilon}{2}(V_{tg}/d_{bg} - V_{bg}/d_{tg})$ applied to BLG, where d_{bg} (d_{bg}) is the thickness of the bottom (top) hBN crystal and ε is its dielectric constant.

Supplementary Fig. 2b shows the top gate voltage dependence of R_a measured in response to 0.13 THz radiation in the dual-gated detector. In the case of zero back gate voltage (black curve), $R_a(V_{tg})$ repeats the behaviour of another detector reported in Fig. 2a of the main text. Namely, the responsivity reaches its maximum of about 200 V/W near the NP where it flips its sign because of the change in the charge carrier type. Note, the absolute value of the maximum responsivity is very close to that reported in the main text (Fig. 2b) highlighting the reproducibility of our detectors' performance. When the back gate voltage is applied, the responsivity increases drastically (red and

blue curves in Supplementary Fig. 2b). Already for a moderate $D \sim 0.1$ V/nm the responsivity increases by more than an order of magnitude and exceeds 3 kV/W. The corresponding noise equivalent power, estimated using the Johnson-Nyquist noise spectral density for the same D, reaches 0.2 pW/Hz$^{1/2}$. This makes our detector competitive not only with other graphene-based THz detectors[1] but also with some commercial semiconductor and superconductor bolometers (Table 1).

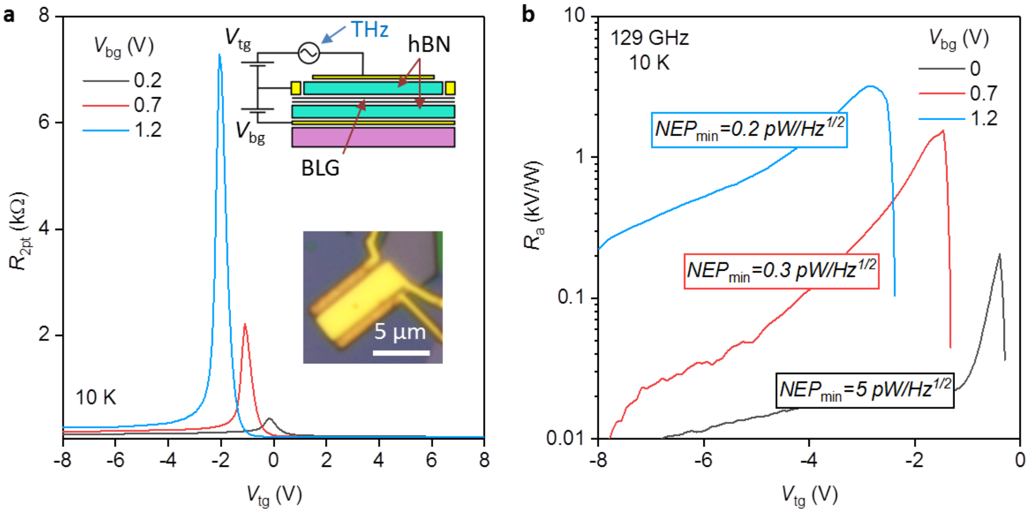

Supplementary Figure 2. **Photoresponse of a dual-gated BLG detector**. **a,** Two-terminal resistance as a function of V_{tg} measured in a dual-gated BLG FET for different V_{bg}. Top inset: Schematic of a dual-gated THz detector. Bottom inset: Optical photographs of the device. **b,** Responsivity as a function of V_{tg} for different V_{bg} measured at given f and T.

Detector	NEP, pW/Hz$^{0.5}$	Operation Temperature, K	Reference
Superconducting hot electron bolometer*	0.1 - 1	2.5 - 4.5	www.boselec.com www.scontel.ru
Semiconductor hot electron bolometer (e.g. InSb)	0.04 - 0.8	1.6 - 4.2	www.infraredlaboratories.com
Dual-gated bilayer graphene THz detector	0.2	10	This work

Supplementary Table 1. **Comparison of cryogenic THz detectors**. *Reported values of NEP were taken for the same frequency (0.13 THz) used to probe our dual-gated devices.

Supplementary Note 3: Further examples of resonant photoresponse

To illustrate that the observed resonant photoresponse is reproducible for different electronic systems embedded in FETs of various lengths L and coupled to different antennas, Supplementary Fig. 3c shows another example of the photovoltage $\Delta U(V_g)$ emerging when the incoming 2 THz radiation is coupled to the broadband logarithmic spiral antenna connected to another FET. The latter is made of BLG having its crystallographic axis aligned with those of hBN, that reveals itself in peculiar three-peaks $R(V_g)$ structure, shown in Supplementary Fig. 3e. The photoresponse curves are rather similar to those shown in Supplementary Fig. 2b of the main text, namely they follow the envelope trend set by the FET-factor $F = -\frac{1}{\sigma}\frac{d\sigma}{dV_g}$ (Supplementary Fig. 3d) superimposed with the resonant peaks. The resonances are periodic in $V_g^{-1/2}$ (inset to Supplementary Fig. 3c) and are clearly seen for both electron and hole doping. Importantly, on the contrary to Fig. 2b, the photoresponse now changes sign multiple times following non-trivial $F(V_g)$ evolution.

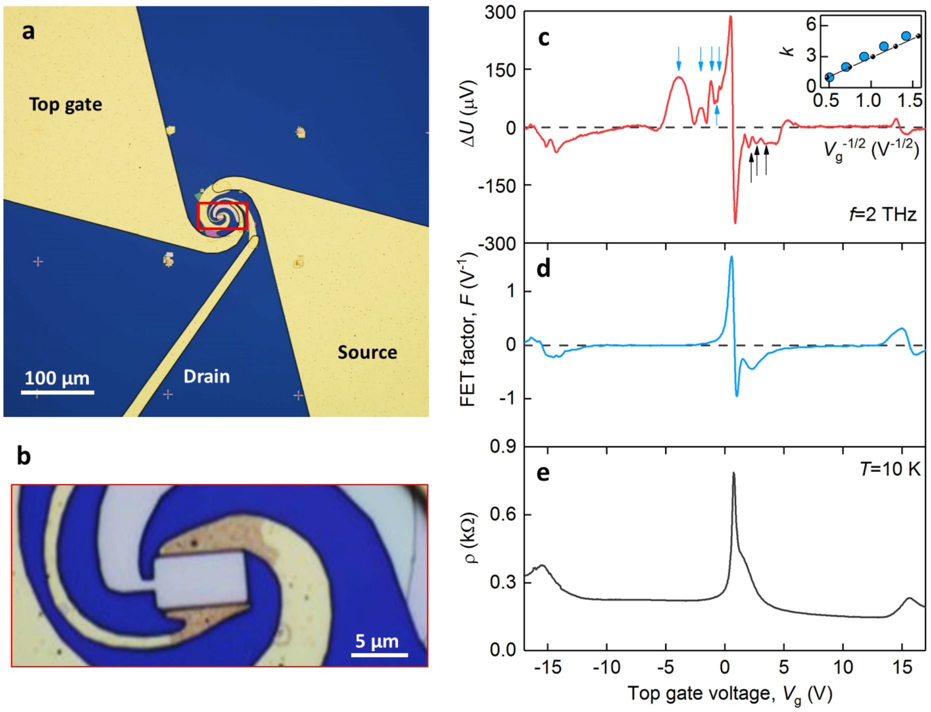

Supplementary Figure 3. **Further examples of resonant photoresponse**. **a-b,** Optical photographs of another THz photodetector. FET channel is coupled to incoming radiation by a broadband logarithmic spiral antenna. The red rectangle in (a) indicates the region shown in (b). **c,** Photovoltage versus V_{g} recorded as a response to $f = 2$ THz radiation in one of our BLG/hBN superlattice devices. Arrows point to the resonant peaks. Inset: Mode number k as a function of $V_{\mathrm{g}}{}^{-1/2}$ taken from the peaks marked by the blue arrows. Black: theoretical dependence expected for $L = 4$ μm, $m = 0.036me$ and $r_{\mathrm{s}}r_{\mathrm{d}} = -1$. **d,** FET-factor F as a function of V_{g} obtained from the data in (a). **e,** Resistivity as a function of V_{g} for the device in (a-b) measured at $T = 10$ K. Three peaks correspond to the secondary neutrality points of BLG/hBN superlattice.

Supplementary Note 4: Resonant detection of sub-THz radiation

We have also studied the performance of our detectors at frequencies intermediate to those reported in Figs. 2a and b of the main text and found that the resonant operation onsets already in the middle of the sub-THz domain. Figure 4 shows the gate voltage dependence of R_{a} recorded in response to 460 GHz radiation. In the vicinity of the charge neutrality point (NP) the responsivity peaks and changes its sign in agreement with the evolution of the FET-factor with the gate voltage (black curve in Supplementary Fig. 4) as discussed in the main text. However, away from the NP the responsivity peaks for both electron and hole doping (stars in Supplementary Fig. 4) despite the fact that $F(V_{\mathrm{g}})$ is featureless. These peaks stem from the plasmon resonances in the FET channel as it follows from the comparison of the experimental data with theory (inset of Supplementary Fig. 4). In good agreement with theory, at lower frequencies the number of resonant modes, which can be observed for the same gate voltage span, is smaller compared to that found at 2 THz (Fig. 2b of the main text). In addition, the resonances appear much broader than those observed at 2 THz (Fig. 2b) which is consistent with the reduced quality factor at sub-THz frequencies.

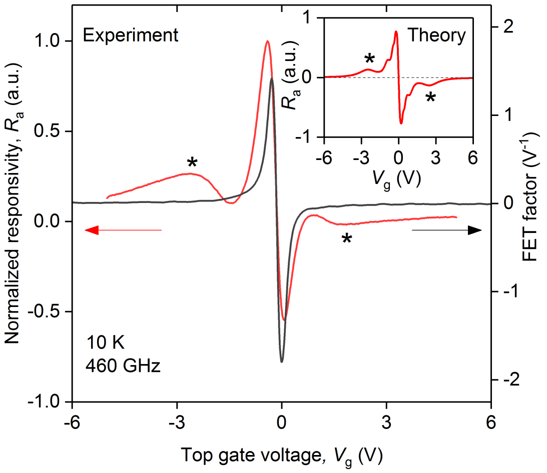

Supplementary Figure 4. **Resonant detection in the sub-THz domain**. Normalized to unity R_{a} as a function of V_{g} measured in response to 460 GHz radiation. Data is acquired on the same device as in Fig. 2 of the main text. Two resonances are clearly visible for electron and hole doping and marked by the black stars. Black curve: FET-factor as a function of V_{tg}. Inset: Theory for $L = 6$ μm, $f = 460$ GHz, $m = 0.036 m_{\mathrm{e}}$, $T = 10$ K and $\tau = 0.6$ ps (eq. 38).

Supplementary Note 5: Fabri-Perot cavity model for plasmonic field-effect transistor

Gated two-dimensional electronic systems support plasma waves with the dispersion relation[2]

$$\omega(\omega + i\tau^{-1}) = \frac{n_0 e^2 q}{2m^* \varepsilon_0 \varepsilon}(1 - e^{-2qd}). \tag{1}$$

where ω and q are the plasmon wavelength and wave vector, respectively, τ is the momentum relaxation time, n_0 is the carrier density, m^* is the effective mass of charge carriers, d it the distance to the gate, ε is the dielectric permittivity, and ε_0 is the vacuum permittivity.

Confinement of a 2d channel by source and drain contacts quantizes the wave vector q and leads to emergence of discrete plasmon frequencies. The quantization conditions can be obtained by requiring the oscillating quantity (e.g. voltage V_ω) to return to its original value after the channel round trip:

$$V_\omega r_s r_d e^{2iqL} = V_\omega, \tag{2}$$

where r_s and r_d are the complex-valued reflection coefficients at the source and drain terminals, respectively. Therefore, eigen frequencies of bounded plasmons can be found from

$$1 - r_s r_d e^{2iqL} = 0. \tag{3}$$

To see that the latter dispersion relation indeed appears in the nonlinear response functions, we model the FET channel as a transmission line (TL) fed by antenna voltage $U_{1\omega} = V_a \cos\omega t$ at the source side[3,4]. The antenna may have finite impedance Z_{a} which will be taken into account at the end of this section. The TL is terminated by load impedance Z_{gd} at the drain side, and by impedance Z_{gs} at the source side. In real device, these impedances are due

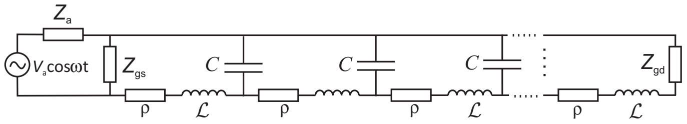

Supplementary Figure 5. **Transmission line equivalent circuit of gated 2d channel.** $\mathcal{L}$ is the kinetic inductance of electrons, ρ is the channel resistivity, C is the effective gate-to-channel capacitance, and $Z_{\rm gd}$ is the load resistance at the drain side. All quantities are measured per unit length of the channel. $Z_{\rm gd} \to \infty$ corresponds to Dyakonov-Shur boundary condition

to the capacitive coupling between the respective electrodes. The TL model is justified by the formal coincidence of TL equations (Telegrapher's equation) with transport equations in a gated FET channel.

The TL elements are specific inductance

$$\mathcal{L} = \frac{m^*}{n_0 e^2 W}, \tag{4}$$

capacitance per unit length

$$C = \frac{2W\varepsilon\varepsilon_0 q}{1 - e^{-2qd}}, \tag{5}$$

and resistance

$$\rho = \mathcal{L}/\tau, \tag{6}$$

where W is the channel width. It is readily seen that the dispersion relation for waves in an infinite transmission line[5]

$$q = \sqrt{C\mathcal{L}}\sqrt{\omega(\omega + \frac{i\rho}{\mathcal{L}})} \tag{7}$$

coincides with plasma wave dispersion (1) with proper values of line parameters (4-6). The characteristic (wave) impedance of transmission line is

$$Z_{\rm tl} = \sqrt{\frac{\omega\mathcal{L} + i\rho}{\omega C}}. \tag{8}$$

A well-known result for current reflection coefficient from a loaded (drain) end of transmission line reads

$$r_d = \frac{Z_{\rm tl} - Z_{\rm gd}}{Z_{\rm tl} + Z_{\rm gd}}, \tag{9}$$

while for source end with fixed voltage

$$r_s = 1. \tag{10}$$

When the reflection coefficients and conditions at the ends of cavity are specified, it is straightforward to write down the solution for voltage across the TL (which is the gate-to-channel voltage in the actual FET):

$$V_\omega(x) = \frac{V_a}{2}\frac{e^{iqx} - r_s r_d e^{-iq(x-2L)}}{1 - r_s r_d e^{2iqL}}, \tag{11}$$

here r_d and r_s are given by Eqs. (9) and (10), respectively. The longitudinal electric field in the channel is given by

$$E_{x\omega} = \frac{qV_a}{2}\frac{e^{iqx} + r_s r_d e^{-iq(x-2L)}}{1 - r_s r_d e^{2iqL}}. \tag{12}$$

As the nonlinear response of the FET is proportional to the properly averaged square of ac electric field in the channel (12), it becomes apparent that responsivity would possess a plasma resonant factor $|1 - r_s r_d e^{2iqL}|^{-2}$, independent of the detection mechanism.

The account of finite antenna impedance results in a a simple "renormalization" of input voltage in Eqs. (11) and (12):

$$V_a \to \frac{V_a}{1+\frac{Z_a}{Z_{\rm gs}\|Z_{\rm tl,in}}}, \tag{13}$$

where $\|$ stands for parallel connection of impedances, and $Z_{\rm tl,in} = Z_{\rm tl}(1-r_d e^{2iqL})/(1+r_d e^{2iqL})$ is the input impedance of the transmission line (we have used $r_s = 1$). It is straightforward to show that the modification of input voltage can be translated in the modification of "resonant denominator"

$$1 - r_d e^{2iqL} \to (1 - r_d e^{2iqL}) \left[1 + \frac{Z_a}{Z_{\rm gs}}\right] + (1 + r_d e^{2iqL}) \frac{Z_a}{Z_{\rm tl}}. \tag{14}$$

The effect of Z_a in the square bracket is the reduction of input voltage due to the drop at internal antenna resistance. Finite value of Z_a in the second term leads to extra broadening or resonances, as analyzed below.

Supplementary Note 6: Gate tuning of graphene plasmons: monolayer vs bilayer

We briefly review the density dependences of plasmon frequencies in single layer graphene (SLG) and bilayer graphene (BLG). The general dispersion relation for gated plasmons in two-dimensional electron system with sheet conductivity σ reads[2]

$$1 + \frac{iq\sigma}{2\omega\epsilon\epsilon_0}(1 - e^{-2qd}) = 0. \tag{15}$$

The study of plasmon dispersions in various two-dimensional systems is therefore reduced to evaluation of their frequency-dependent conductivity. In the classical ($\hbar\omega \ll \varepsilon_F$) long-wavelength ($q \ll \omega/v_F$) limit, the latter is found from the Boltzmann equation[6]

$$\sigma = \frac{e^2}{2} \int\limits_0^{+\infty} d\varepsilon \rho(\varepsilon) \frac{v_p^2}{-i\omega + \tau_p^{-1}} \left(-\frac{\partial f_0}{\partial \varepsilon}\right), \tag{16}$$

where $\rho(\varepsilon)$ is the density of states, v_p is the velocity of carrier with momentum p, and f_0 is the equilibrium distribution function. In case of BLG, $\rho(\varepsilon) = 2m/\pi\hbar^2$, $v_p = p/m$, which results in ordinary Drude conductivity

$$\sigma_{\rm BLG} = \frac{ne^2\tau_p/m}{1 - i\omega\tau_p}. \tag{17}$$

In case of SLG, $\rho(\varepsilon) = 2\varepsilon/\pi\hbar^2 v_F^2$, $v_p = v_F$, and the conductivity reads

$$\sigma_{\rm SLG} = \frac{e^2}{\pi\hbar^2} \frac{kT \ln(1 + e^{\varepsilon_F/kT})}{-i\omega + \tau_p^{-1}} \approx \frac{e^2}{\pi\hbar^2} \frac{\varepsilon_F}{-i\omega + \tau_p^{-1}}. \tag{18}$$

The latter equality is valid at low temperatures. Using the low-temperature relation between density and Fermi energy in SLG $n = \varepsilon_F^2/p\hbar^2 v_F^2$, we readily observe that classical conductivity of SLG is still given by the Drude formula (17) with density-dependent mass $m \to \varepsilon_F/v_F^2 \propto n^{1/2}$. Combining Eqs. (15), (17) and 18, we observe that plasma frequency in BLG scales as $\omega \propto n^{1/2}$, while in SLG $\omega \propto n^{1/4}$.

Supplementary Note 7: Resonance broadening and plasmon lifetime

Before discussing the physics beyond THz rectification, we specify mechanism-independent quantities, namely, the positions of plasma resonances and resonance width. Introducing the complex reflection phase

$$\exp\left[i\theta_r' - \theta_r''\right] = -r_s r_d, \tag{19}$$

we transform the "resonant denominator" in eq. (1) of the main text

$$R(\omega, V_g) \propto |1 - r_s r_d e^{2iqL}|^{-2} = \frac{1}{2} \frac{e^{\theta_r'' + 2q''L}}{\cosh(\theta_r'' + 2q''L) + \cos(\theta_r' + 2q'L)}, \tag{20}$$

The maxima of responsivity correspond to wave vectors

$$q_0' = \frac{\pi}{2L}(2k + 1 + \theta_r'/\pi). \tag{21}$$

In the case of Dyakonov-Shur boundary conditions realized in our devices, $\theta_r = 0$, and the first resonance corresponds to L equal to the quarter of plasmon wavelength. Assuming reflection and scattering losses to be small, the lineshape (20) can be transformed to Lorentzian in the vicinity of each peak

$$R(\omega, V_g) \propto \frac{e^{\theta_r'' + 2q''L}}{(q''L + \theta_r''/2)^2 + (q' - q_0)^2 L^2}. \tag{22}$$

The full width at half-height is given by

$$\frac{\delta q}{q_0} = \frac{1}{\omega\tau} + \frac{2}{\pi}\frac{\ln|r_s r_d|^{-1}}{2k+1} \equiv \frac{1}{\omega\tau_p}, \tag{23}$$

here we have introduced the plasmon lifetime τ_p which is below the scattering time τ_p due to resonator loss. It is also possible to take into account the effect of finite antenna resistance on plasmon linewidth. To this end, one should transform resonant denominator of the form (14) in the vicinity of resonance. This leads us to

$$\frac{1}{\omega\tau_p} = \frac{1}{\omega\tau} + \frac{2}{\pi}\frac{\ln|r_s r_d|^{-1} + 2Z_{\rm a}'/Z_{\rm tl}'}{2k+1}. \tag{24}$$

The above equation clearly demonstrates that inverse plasmon lifetime $\tau_{\rm p}^{-1}$ is the sum of electron momentum relaxation rate τ^{-1}, contact damping rate

$$\tau_{\rm cont}^{-1} = \frac{2\omega}{\pi}\frac{\ln|r_s r_d|^{-1}}{2k+1} = \frac{s}{L}\ln|r_s r_d|^{-1} \propto \sqrt{V_g}, \tag{25}$$

and damping rate due to antenna resistance

$$\tau_{\rm ant}^{-1} = \frac{2s}{L}\frac{Z_{\rm a}'}{Z_{\rm tl}'} \propto V_g. \tag{26}$$

The two latter contributions to damping rate are minimized in the vicinity of charge neutrality point. Examples of calculated plasmon lifetime including the contributions of contacts and antenna are shown in Supplementary Fig. 6. Effects of radiative contribution to plasmon damping on detector responsivity is shown in Supplementary Note 9, along with the discussion of detection mechanisms.

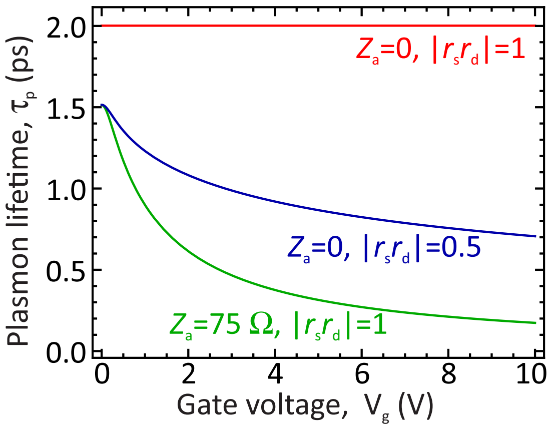

Supplementary Figure 6. **Plasmon damping.** Calculated plasmon lifetimes assuming momentum relaxation time $\tau = 2$ ps and no antenna and contact losses (red), lossless antenna and reflection coefficient $|r_s r_d| = 0.5$ (blue), antenna resistance $Z_a' = 75$ Ohm and perfect reflection (green). Plasmon velocity s at charge neutrality point is limited by residual carriers with density $n^* = 5 \times 10^{10}$ cm^{-2}.

As the wave vector at fixed frequency is inversely proportional to wave velocity, $q = \omega/s \propto V_g^{-1/2}$, expression (23) can be transformed to the voltage scale

$$\frac{\delta V_g^{-1/2}}{V_g^{-1/2}} = \frac{1}{\omega\tau_p}. \tag{27}$$

Supplementary Note 8: Photothermoelectric rectification in Fabri-Perot cavity

Asymmteric feeding of THz radiation results in asymmetric heating of the device and emergence of thermoelectric effect. The resulting dc voltage is[7]

$$eV_{\rm pte} = (S_{ch} - S_{cont})\left[T_s - T_d\right], \tag{28}$$

where S_{ch} is the Seebeck coefficient in the gated channel, and S_{cont} – in the metal-doped graphene contact, T_s is the local temperature at the source junction and T_d is at the drain junction . From now on, we refer to the gated part of graphene as "channel" and ungated part – as "contact". The doping of ungated part does not depend on gate voltage, however, it can be non-uniform due to the effects of built-in field near metal contacts.

The temperature difference $T_s - T_d$ induced by non-uniform heating of the device can be found from the solution of heat transfer equation in the channel:

$$\frac{\partial^2 T}{\partial x^2} + \frac{T - T_0}{L_T^2} = -\frac{q(x)}{\chi_{ch}}, \tag{29}$$

$$\tag{30}$$

where $q(x) = 2{\rm Re}\sigma_\omega |E_{x\omega}|^2$ is the Joule heating power, χ_{ch} is the electron thermal conductivity in the channel, $L_T = (\chi_{ch}\tau_\varepsilon/C_e)^{1/2}$ is the thermal relaxation length, τ_ε is the energy relaxation time due to heat sink into substrate phonons[8], and C_e is the heat capacitance of the electronic system. Equation (29) is supplemented by the boundary conditions at the boundaries of gated domain

$$\frac{\chi_{cont}}{L_{cont}}(T_s - T_0) = \chi_{ch}\nabla T_s, \qquad \frac{\chi_{cont}}{L_{cont}}(T_d - T_0) = -\chi_{ch}\nabla T_d; \tag{31}$$

these conditions follow from the continuity of heat flux at the interfaces. The sought-for temperature difference between source and drain can be obtained in the closed form under the following simplifying assumptions (1) the Joule heating occurs only in the channel (2) the temperature drop across the contacts is much less than maximum overheating in the channel. Both conditions are justified by the small length of the contacts $L_{cont} \ll L$. Under these assumptions, the expression for the photo-thermoelectric voltage acquires a physically appealing form

$$eV_{\rm pte} = (S_{ch} - S_{cont})\frac{l_{cont}}{\chi_{cont}}\int_0^L 2{\rm Re}\sigma_\omega |E_{x\omega}|^2 \frac{\sinh\frac{x-L/2}{L_T}}{\sinh\frac{L}{2L_T}}dx. \tag{32}$$

The quantity χ_{cont}/l_{cont} is the thermal *conductance* of the contact, while the integral is the difference of heat fluxes traveling toward the source and toward the drain. The kernel of the integral is anti-symmetric with respect to the middle of the channel $x = L/2$, therefore, the PTE signal appears only due to asymmetric heating $q(x)$. Final evaluation of PTE voltage is performed by substituting the solution for electric field (12) into (32):

$$eV_{\rm pte} = (S_{ch} - S_{cont})\frac{2L_{cont}L_T{\rm Re}\sigma_\omega}{\chi_{cont}}\frac{|q|^2 V_a^2}{e^{q''L+\theta_r''/2}|1 - r_s r_d e^{2iqL}|^2}\times$$
$$\left[\sin 2\alpha' \frac{2q'L_T\cos q'L - \coth\frac{L}{2L_T}\sin q'L}{1+(2q'L_T)^2} + \sinh 2\alpha'' \frac{2q''L_T\cosh q''L - \coth\frac{L}{2L_T}\sinh q''L}{1-(2q''L_T)^2}\right], \tag{33}$$

where $\alpha = \theta_r + qL/2$.

Supplementary Note 9: Dyakonov-Shur rectification in Fabri-Perot cavity

The so-called Dyakonov-Shur rectification includes two physically different nonlinearities. One contribution to the rectified current appears due to simultaneous modulation of 2d channel conductivity and application of longitudinal field. This effect, also known as resistive self-mixing, results in the rectified voltage

$$V_{\rm rsm} = 2{\rm Re}\int_0^L \frac{1}{\sigma_{\omega=0}}\frac{d\sigma_\omega}{dV_{\rm gc}}V_{-\omega}(x)\frac{\partial V_\omega(x)}{\partial x}dx. \tag{34}$$

Here $\sigma_{\omega=0} = ne^2\tau/m$ is the dc conductivity of a 2D channel, n is the carrier density, e and m are the elementary charge and effective mass of charge carriers respectively, τ is the momentum relaxation time, and $\sigma_\omega = \sigma_{\omega=0}/(1-i\omega\tau)$ is the high-frequency conductivity. Evaluation of the integral leads us to the result

$$V_{\rm rsm} = \frac{V_a^2/V_g}{\sqrt{1+\omega^2\tau^2}}\frac{\frac{q''}{q'}[\cos\theta_r' - \cos(\theta_r' + 2q'L)] + \frac{q'}{q''}[\cosh\theta_r'' - \cosh(\theta_r'' + 2k''L)]}{2e^{\theta_r''+2q''L}|1 - r_s r_d e^{2iqL}|^2} \tag{35}$$

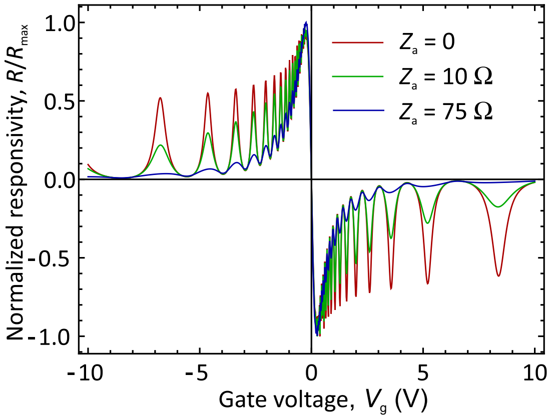

Supplementary Figure 7. **Dyakonov-Shur photoresponse.** Calculated Dyakonov-Shur responsivity of BLG FET detector as a function of gate voltage at different values of antenna impedance. All curves are normalized by their maximum value reached in the vicinity of NP. Parameters: channel length $L = 6$ μm, momentum relaxation time $\tau = 2$ ps, radiation frequency $f = 2$ THz, gate-channel separation $d = 80$ nm, residual carrier density $n^* = 5 \times 10^{10}$ cm^{-2}.

Another contribution to rectified voltage stems from the difference of kinetic energies of electron fluid at the source and drain side (Bernoulli law). The underlying nonlinearity is manifested by convective term $(\mathbf{u}\nabla)\mathbf{u}$ in the Euler equation for electron fluid[9]. The corresponding rectified voltage is

$$eV_{\rm nl} = \frac{e^2}{m^*(\omega^2+\tau^{-2})}\left[|E_{x\omega}(L)|^2 - |E_{x\omega}(0)|^2\right], \tag{36}$$

where $E_{x\omega}$ is the complex amplitude of high-frequency longitudinal field in the channel given by (12). Using the result for electric field (12), we find

$$V_{\rm nl} = \frac{1}{4}\frac{V_a^2/V_g}{\sqrt{1+\omega^{-2}\tau^{-2}}}\frac{\left|1+r_s r_d e^{2iqL}\right|^2 - \left|(1+r_s r_d)e^{iqL}\right|^2}{\left|1-r_s r_d e^{2iqL}\right|^2}. \tag{37}$$

Equations (35) and (37) generalize the known results of Dyakonov and Shur for arbitrary loading of the plasmonic FET at the terminals. Naturally, they reduce to the result of Ref. 9 for high-impedance drain load $\theta_r = 0$, yielding the photovoltage given by:

$$\Delta U = -\frac{1}{4}\frac{V_a^2}{V_g}\left[1+\frac{2}{\sqrt{1+(\omega\tau)^{-2}}} - \frac{1+\frac{2\cos 2q'L}{\sqrt{1+(\omega\tau)^{-2}}}}{\sinh^2 q''L+\cos^2 q'L}\right], \tag{38}$$

The factor in square brackets peaks when the length of the FET channel matches odd multiples of the plasmon quarter-wavelength.

We note that Eqs. (35), (37) and (38) diverge as the dc gate voltage V_g tends to zero. In fact, this divergence stems from the gradual-channel approximation, relating carrier density and gate voltage $CV_g = en$, that fails near the charge neutrality point. The account of ambipolar transport involving electrons and holes leads to a simple replacement in Eqs. (35), (37) and (38):

$$\frac{1}{V_g} \to \frac{n/m_n^2 - p/m_p^2}{n/m_n + p/m_p}\frac{1}{s^2}, \tag{39}$$

where n and p are electron and hole densities, m_n and m_p are their effective masses, and $s^2 = (n/m_n + p/m_p)e^2/C$ is the plasma wave velocity in ambipolar system.

In the main text, the experimental photoresponse was compared with the DS photovoltage from eq. (38) corrected by eq. (39). The full picture of calculated Dyakonov-Shur photovoltage vs gate voltage is shown in Supplementary Fig. 7. Along with the result for perfect reflection from the drain and ideal voltage source ($Z_{\rm a} = 0$, red line), it also shows the effect of finite antenna impedance on resonance width (green and blue lines). In accordance with the discussed antenna-induced "renormalization" of input voltage, eq. 13, the resonances at high carrier density are highly broadened due to finite $Z_{\rm a}$. The width of resonances at low density, on the contrary, is mainly determined by momentum relaxation time.

Supplementary Note 10: Detector operation outside the linear regime

The data reported in the main text were obtained in the regime where the detector's photovoltage grew linearly with the power P of incoming radiation. With increasing P outside the linear regime, the heating of graphene's electronic system by high-frequency ac capacitive currents flowing between the source and gate terminals may affect the resulting responsivity. In order to reveal the role of heating, we have studied the response of our detectors at varying P and found that outside the linear regime, R_a decreases with increasing P. We attribute this decrease to the modification of the channel conductivity with increasing electronic temperature. To support this statement, we plot the FET-factor obtained by measuring the samples conductivity at different T inside the sample chamber. Clearly, R_a acquired at different P follows the evolution of the FET-factor F with T. This is reflected in the shift and decrease of the responsivity extrema with increasing P. We also refer to Fig. 2a of the main text which shows $R_a(V_g)$ at different T that resembles the behaviour shown in Supplementary Fig. 8.

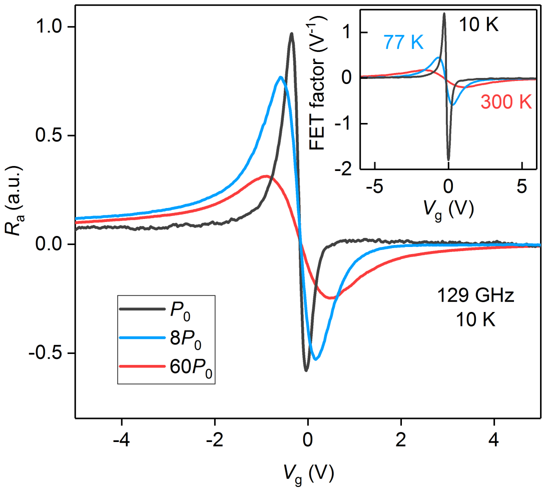

Supplementary Figure 8. **Role of electron heating.** Normalized to unity responsivity as a function gate voltage measured in one of our BLG detectors at given T and f for different P. $P_0 = 4$ μW. Inset: FET-factor as a function of V_g for different T.

Supplementary References

[1] Koppens, F. H. L. *et al.* Photodetectors based on graphene, other two-dimensional materials and hybrid systems. *Nature Nanotechnology* **9**, 780–793 (2014).

[2] Chaplik, A. Possible crystallization of charge carriers in low-density inversion layers. *Sov. Phys. JETP* **35**, 395 (1972).

[3] Aizin, G. R. & Dyer, G. C. Transmission line theory of collective plasma excitations in periodic two-dimensional electron systems: Finite plasmonic crystals and tamm states. *Phys. Rev. B* **86**, 235316 (2012).

[4] Dyer, G. C. *et al.* Induced transparency by coupling of tamm and defect states in tunable terahertz plasmonic crystals. *Nature Photonics* **7**, 925 (2013).

[5] Collin, R. E. *Field theory of guided waves* (McGraw-Hill, 1960).

[6] Das Sarma, S., Adam, S., Hwang, E. H. & Rossi, E. Electronic transport in two-dimensional graphene. *Rev. Mod. Phys.* **83**, 407–470 (2011).

[7] Bandurin, D. A. *et al.* Dual origin of room temperature sub-terahertz photoresponse in graphene field effect transistors. *Applied Physics Letters* **112**, 141101 (2018).

[8] Low, T., Perebeinos, V., Kim, R., Freitag, M. & Avouris, P. Cooling of photoexcited carriers in graphene by internal and substrate phonons. *Phys. Rev. B* **86**, 045413 (2012).

[9] Dyakonov, M. & Shur, M. Detection, mixing, and frequency multiplication of terahertz radiation by two-dimensional electronic fluid. *IEEE Transactions on Electron Devices* **43**, 380–387 (1996).
